# Antimicrobial Stewardship in Healthcare: Exploring the Role of Nurses in Promoting Change, Identifying Barrier Elements and Facilitators—A Meta-Synthesis †

**DOI:** 10.3390/healthcare12212122

**Published:** 2024-10-24

**Authors:** Antonio Bonacaro, Francesca Giovanna Solfrizzo, Domenico Regano, Fabio Negrello, Celeste Domeniconi, Alessandra Volpon, Silvia Taurchini, Paola Toselli, Consuelo Baesti

**Affiliations:** 1 Department of Medicine and Surgery, University of Parma, Via Gramsci 14, 43125 Parma, Italy; 2 IRCCS Ospedale San Raffaele, Via Olgettina 60, 20132 Milano, Italy; solfrizzo.francesca@hsr.it; 3 IRCCS Azienda Ospedaliero, Universitaria di Bologna, Via Albertoni 15, 40138 Bologna, Italy; domenico.regano@unibo.it (D.R.); fabio.negrello@aosp.bo.it (F.N.); 4 Ausl Romagna, Via A. De Gasperi 8, 48121 Ravenna, Italy; celeste.domeniconi91@gmail.com (C.D.); consuelo.baesti@studenti.unipr.it (C.B.); 5 Azienda ULSS 1 Dolomiti, Via Feltre 57, 32100 Belluno, Italy; alessandra.volpon@gmail.com; 6 ASL Roma 4, Via Terme di Traiano 39/A, 00053 Civitavecchia, Italy; silvia.taurchini@studenti.unipr.it; 7 Azienda Ospedaliera Universitaria Santi Antonio e Biagio e Cesare Arrigio, Via Venezia 16, 15121 Alessandria, Italy; toselli63@gmail.com

**Keywords:** antimicrobial stewardship (AMS), barriers, facilitators, nurse role, antibiotic use, training, organization

## Abstract

Background: Antimicrobial stewardship (AMS) involves a coordinated set of actions aimed at promoting the appropriate use of antibiotics within healthcare settings. This systematic review of qualitative studies assessed nurses’ knowledge and perceptions of the barriers and facilitators that impact their involvement in AMS programs. Methods: This meta-synthesis followed the Joanna Briggs Institute methodology for systematic reviews of qualitative evidence. Relevant studies published between 2018 and 2023 were identified through searches on PubMed, CINAHL, EMBASE, PsycINFO, and Google Scholar. The studies were critically appraised using the CASP checklist, with 19 articles meeting the inclusion criteria from five continents. Results: Six recurring themes emerged from the analysis of nurses’ experiences and opinions regarding their roles in AMS programs. These themes included the organization of AMS programs, availability of resources, training and education, communication, and the evolving role of nurses in AMS. Conclusions: Nurses at every level of the profession might play a crucial role in antimicrobial stewardship. Although active involvement of nurses in antibiotic stewardship requires further exploration and research, this topic is being examined internationally. The literature on this subject primarily analyzes the phenomenon from a quantitative perspective rather than a qualitative one, and it is contextualized more within hospital settings rather than community settings.

## 1. Introduction

Antimicrobial stewardship (AMS) refers to a coordinated set of context- and time-specific actions aimed at promoting the appropriate and responsible use of antibiotics in healthcare settings [1]. In Italy, the proportion of antibiotic-resistant infections increased from 17% in 2005 to 30% in 2015, with projections estimating it may reach 32% by 2030 [2]. Across Europe, 671,689 healthcare-associated infections (HAIs) were identified in 2015, of which 33,110 were fatal [3]. Approximately one-third of these HAIs occurred in Italy, underscoring the urgent need for improved monitoring and management of antibiotic use [3]. In Italy, national efforts to combat antimicrobial resistance (AMR) are guided by the National Plan Against Antibiotic Resistance (PNCAR) for 2022–2025 [4]. Over the past fifteen years, growing global concerns about AMR have prompted the creation of guidelines for developing AMS programs by various scientific societies [5]. These guidelines recommend the involvement of healthcare professionals, including infectious disease specialists, pharmacists, microbiologists, and infection control managers, in stewardship efforts [6]. Recently, the participation of nurses in AMS programs has gained attention, though their role remains poorly defined and marginal [7].

Nurses are primarily involved in AMS programs for epidemiological data collection or ensuring proper collection and storage of microbiology samples. However, many nurses view their involvement in AMS as an opportunity to enhance the value of their existing roles, rather than as an additional burden [8]. Expanding the leadership roles of nurses within AMS programs could potentially enhance the impact of these programs and send a strong message to policymakers about the importance of their implementation and adoption.

The aim of this meta-synthesis was to assess nurses’ knowledge and perceptions of the factors that influence their involvement in AMS programs. A preliminary search of the literature revealed increasing engagement of nurses in AMS, despite a healthcare model that predominantly centers on physicians. This study explored the barriers and facilitators influencing nurses’ empowerment and participation in AMS, highlighting the critical role nurses play in the fight against antibiotic resistance [9].

## 2. Materials and Methods

This meta-synthesis was conducted in accordance with a research protocol registered in PROSPERO (registration number CRD42023460278). The systematic review was performed in the light of the Joanna Briggs Institute (JBI) methodology for systematic reviews of qualitative evidence [10].

The following research question was formulated to develop the research protocol:

*‘What are the experiences, attitudes, and competencies of nurses with regard to antimicrobial stewardship, and what are the facilitators and barriers within the healthcare context in establishing a nursing role in antimicrobial stewardship programs?’*

This review considered studies that explored the experiences, perceptions, competencies, and knowledge of nurses to highlight strengths and potential barriers that hinder the involvement of nurses in antimicrobial stewardship management.

A preliminary search was conducted to identify any gaps in the existing literature and to establish a solid foundation for selecting studies to be included in the meta-synthesis. The text words in the titles and abstracts of articles meeting the search criteria, along with index terms used to describe the articles, were utilized to develop a comprehensive search string. The search string included all identified keywords and index terms and was tailored for each source of information included. The identified keywords were: “*antimicrobial stewardship*”, “*antibiotic stewardship program*”, “*nurses*”, “*nursing*”, “*nurses role*”, “*qualitative*”, “*experience*”, “*barrier*”, and “*facilitators*”.

The final search terms were associated by using the SPIDER search tool and combined through the Boolean operators AND and OR [11].

S (sample): Nurs*
PI (phenomenon of interest): Antimicrobial Stewardship
D (design): Focus group, observation, interview
E (evalutation): Knowledge, Barriers, Facilitators, Attitudes, Perceived attitudes, Perceived barriers
R (research type): All qualitative studies

The databases used were PubMed, CINAHL, PsycINFO, EMBASE and Google Scholar.

With the formulation of the following search string for PubMed:

(nurs*) AND (“Antimicrobial Stewardship”[Mesh] OR “antibiotic stewardship programmes”) AND (“Knowledge”[Mesh] OR “Health Knowledge, Attitudes, Practice”[Mesh] OR “Attitude”[Mesh] OR barrier* OR facilitator* OR “perceived attitudes” OR “perceived barriers”) AND (“Qualitative Research”[Mesh] OR “qualitative study”).

With the formulation of the following search string for CINAHL, PsycINFO, EMBASE and Google Scholar: (nurs*) AND (“Antimicrobial Stewardship” OR “antibiotic stewardship programmes”) AND (Knowledge OR “Health Knowledge, Attitudes, Practice” OR Attitude OR barrier* OR facilitator* OR “perceived attitudes” OR “perceived barriers”) AND (“Qualitative Research” OR “qualitative study”).

Studies from 2018 to 2023 in English and Italian were considered; studies published in other languages were excluded due to difficulties in conducting an accurate translation. All healthcare settings were included without applying any restriction based on country, healthcare organizational model, or professional profile. Since this systematic literature search focused on adult patients, studies involving pediatric nurses were excluded.

### 2.1. Study Selection

A total of 577 studies were identified after the initial search. After removing 77 duplicates and screening titles and abstracts, 468 studies were excluded. Full-text reviews of the remaining studies resulted in 19 articles meeting the inclusion criteria. The inclusion criteria were set to consider studies published within the last five years, written in English or Italian. Studies in other languages were excluded due to limitations in reliable translation capabilities within the review team. We also considered studies from all healthcare settings without restrictions based on the healthcare model, country, or professional profile. However, pediatric nurses were excluded from the review due to the specialized nature of their expertise, which does not generalize to the broader population of healthcare workers involved in antimicrobial stewardship. Furthermore, studies categorized as rapid reviews and study previews were excluded. The exclusion of rapid reviews was based on concerns regarding the depth of methodological rigor. As rapid reviews often condense the systematic review process, they may not offer the comprehensive data required for our meta-synthesis. Similarly, study previews were excluded as they frequently lack complete datasets and analysis, which are crucial for the reliability and validity of the synthesis.

**Figure 1:**
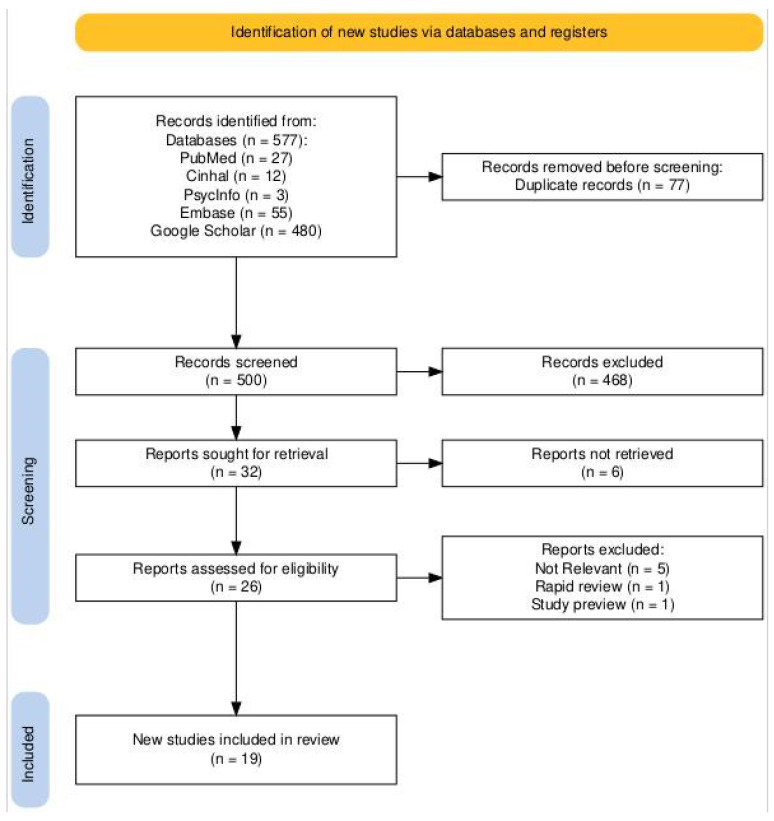
Prisma Statement.

The study selection process is illustrated using a Preferred Reporting Items for Systematic Reviews and Meta-Analyses (PRISMA) flowchart [12] (Figure 1).

### 2.2. Methodological Quality Assessment

The 19 eligible studies were critically appraised by two independent reviewers using the CASP (Critical Appraisal Skills Program) checklist for qualitative research [13]. Of these studies, 15 met all nine critical appraisal criteria.

Two studies met eight out of nine criteria due to a lack of clarity in the researcher–participant relationship [14,15]. One study lacked ethics committee approval, meeting only eight out of nine criteria [16].

A third reviewer was consulted for one study to resolve questions surrounding its methodological clarity, particularly in sampling and researcher–participant interactions. This study was included with reservations [17].

### 2.3. Data Extraction and Synthesis

Data were extracted from the included studies using a tool developed by the reviewers. The data included the characteristics of the participants, their roles in AMS programs, the study setting, and the barriers and facilitators influencing AMS program implementation (see Supplementary Materials). The results were synthesized using the JBI Manual for Evidence Synthesis [10], which allowed for aggregation and synthesis of the findings into categories representing the meta-synthesis. Each study was thoroughly reviewed by the research team to ensure an in-depth understanding of the collected materials. The general themes identified by the authors were classified into sub-themes, which were supported by the study findings. The results were then synthesized to generate a set of unambiguous, credible conclusions (see Table 1).

### 2.4. Evaluation of Data Confidence

The synthesized results were assessed using the ConQual approach, which establishes confidence in the output of qualitative research synthesis [18]. Each finding was summarized, and the associated ConQual score was developed based on reliability and credibility. A summary of the results, including the main elements of the review and the associated ConQual score, is presented in Table 2. Discrepancies among reviewers were resolved through discussion.

## 3. Results

The analysis identified six meta-syntheses based on the studies regarding nurses’ experiences and perceptions of AMS programs. These meta-syntheses are summarized in Table 3.

**Figure 2:**
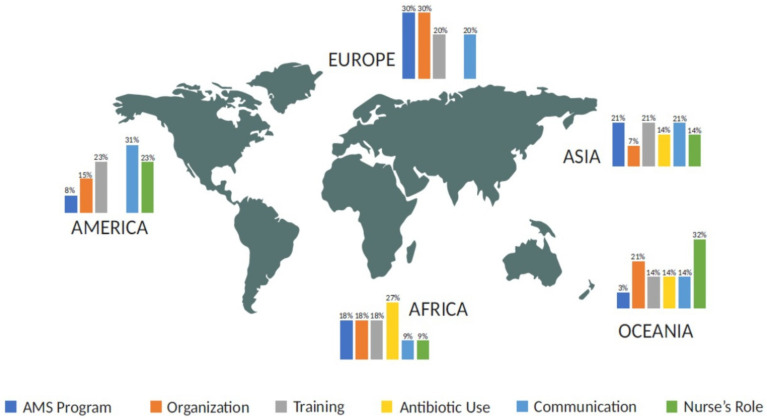
Meta-synthesis distribution of the examined literature.

The results are contextualized by geographical area, highlighting the global variation in AMS program implementation as presented in Figure 2.

**AMS Programs**
*Adherence to AMS programs*

Ten findings (10 U) defining this category were combined. The nurses interviewed cited lack of collaboration, non-compliance, and absence of feedback on AMS activities in their respective units as key factors for non-adherence to AMS programs. One facilitator identified was the assignment of leadership roles to nurses within AMS teams. In fact, a participant stated,

*“[…] I think the doctors are on board, they know the guidelines, they use the guidelines, but sometimes they’re overruled”*.

(P019) [19]

*Nurses’ perceptions of their role in AMS*

Nurses did not always perceive the need for AMS education and training (AMS E&T) or understand its importance in improving antibiotic prescribing practices. One interviewee remarked,

*“I’m just at ward level giving out the antibiotics that are prescribed”*

(FG 14 nurses) [20]

*Nurses’ Role in AMS from the Perspective of Other Healthcare Professionals*

The studies indicated that physicians and pharmacists were seen as predominant players in AMS programs, while the role of nurses was primarily focused on direct patient care. One nurse leader stated,

*“Nurses focus on nursing care but do not have a direct role in prescribing antibiotics. So, taking a role in AMS is quite difficult”*

(Nurse Leader 1) [21]

**Organization, context and resources**

This category, defined by fourteen findings (11 U, 3 C), highlights several organizational challenges and resource-related issues impacting AMS implementation.

*Workload*

Many nurses interviewed reported that workload, combined with the complexity of tasks, is a significant risk factor for non-adherence to protocols. This challenge is exacerbated by documentation processes that are not user-friendly or easily applied in practice.

*“We still do (preparing more antibiotics at once) because we keep them in the fridge, but it’s not recommended. We try to relieve ourselves because of workload so we end up doing shortcuts”*

(Male nurse 12, female ward) [22]

*Material and economic resources*

Some nurses highlighted the lack of Information Technology (IT) tools aimed at promoting a fast and smooth workflow and transmission of clinical information for healthcare personnel.

*“Currently we have to track laboratory results manually; that would be much easier for nurses if the IT (Information Technology) system could pop up the results automatically especially in cases of patients with serious infection”*

(Nurse leader 5) [21]

*Transmission modes of information*

The literature review revealed that the communication methods related to AMS protocols and procedures are often unclear and not timely.

*“I think the algorithms and clinical pathways work, definitely. But when there’s an overload of information—pages and pages of stuff—it can become sensory overload. There are so many protocols and procedures that it becomes overwhelming, and people just start ignoring them, which makes them ineffective”*

(RN 5) [23]

*“We have ongoing reminders, weekly emails, and team meetings to review our progress. There are screensavers on our computers and posters everywhere. Patient handouts are part of the template, but they are separate and on the desktop. We also have self-learning training modules”*

(Nurse Practitioner) [24]

*Accreditation Standards*

One study indicated that the need to meet national accreditation standards serve as a motivating factor for adherence to AMS programs.

*“In aged care, the only way things get followed up on is when they’re included in the accreditation standards—100%”*

(Nurse 10) [25]

*Promotion of Multi-Professional Working and Advanced Training for Nurses*

In a study conducted in France, nurses stressed the importance of teamwork and advanced training to promote active and competent participation in AMS programs.

*“We’re currently considering advanced practice registered nurses. It seems logical that those focusing on pharmacology-related functions could take on transversal roles within their institutions”*

(NHN 19) [26]

*Legislative support*

Some studies emphasized the importance of legislative support in enacting regulations that define the necessary competencies for healthcare professionals.

*“Legally, something should perhaps be changed. It needs to be incorporated into the decree of competence”*

(NHN 20) [26]

**Training, Knowledge and Education**

Thirteen findings (13 U) highlighted the importance of education and training in AMS.

*University Training*

Several nurses pointed out the lack of dedicated infection prevention control (IPC) training in university programs. According to the interviewees, this gap leaves newly graduated professionals feeling inadequately prepared for the clinical realities they face.

*“We’re not informed or trained enough on antibiotics, for example. Since I graduated, I learned about antibiotics in school, but we’re not updated on new antibiotics or those that are less commonly used. We find out as we use them”*

(NHN 10) [26]

*Postgraduate Training*

Other respondents highlighted how insufficient and inconsistent education contributes to poor adherence to protocols aimed at combating antibiotic resistance.

*“When you first come to work in the mornings, I don’t see many people washing their hands”*

(N6) [15]

*“We are agreeable to go for courses to understand about the drugs [referring to antibiotics]. So that when the doctors order, we understand the rationale behind it. If it’s something not useful, we can always say that we learned it from this course [that]. I don’t think this drug will be useful”.*

(FGD013, SGH) [6]

*Nurses’ Knowledge of Antibiotics and the Influence in AMS*

The literature consistently reports a lack of knowledge among nurses regarding antibiotics. In some cases, the infectious risk is not perceived as an urgent issue.

*“I work in orthopedics, and we see a lot of resistance, even more in the past 12 to 24 months... resistance to multiple things, and other infections in patients’ wounds” (NUM 2, FG3). “NUM2: I remember when we first encountered our first VRE patient on the orthopedic unit—it was probably 9 or 10 years ago. Now, it’s like everyone seems to have VRE” (NUM3). “NUM3: I know! It’s like the boy who cried wolf. It doesn’t shock you anymore”*

(FG3) [14]

*Training through Guidelines*

Interviewees emphasized that guidelines can help support nurses’ critical thinking skills.

*“NUM3: A lot of doctors don’t read the guidelines. Even if they do, they still stick to their own practice. It’s a culture, and no one is enforcing any rules. NUM2: Yes, they can do whatever they want, basically”*

(NUM 3) [14]

**Use of antibiotics**

Nine findings (9 U) focused on the role of antibiotics in AMS.

*Antibiotic Prescription*

Due to medical staff shortages, nurses are increasingly taking on responsibilities related to antibiotic administration and management, even in the absence of legislative frameworks. Many nurses, with their close involvement in patient care, expressed concerns about the timeliness and appropriateness of antibiotic prescriptions.

*“Based on what patients report about signs and symptoms and my experience observing the doctor, combined with internet searches, I sometimes give antibiotics”*

(Female, 35, nurse) [27]

*Rapid Access to Antibiotics*

Nurses often expressed surprise at the level of awareness patients have regarding antibiotics. The perception that antibiotics offer a quick solution to problems is deeply ingrained in the population, with unregulated access to antibiotics worsening the issue in some countries.

*“Such medications are sold everywhere. You can just go to the pharmacy and tell them you need this or that antibiotic and you will have it”*

(Nurse) [17]

*Benchmarking*

Only one study demonstrated how benchmarking can lead to an in-depth analysis of prescription data, allowing adjustments to treatment goals and care standards.

*“We’ve reduced antimicrobial usage by 80% just by reviewing long-term antimicrobials and evaluating who is on what and why”*

(Nurse 10) [25]

**Communication and Relationship**

Fourteen findings (12 U, 2 C) addressed the importance of communication and collaboration.

*Professional Communication*

Nurses emphasized the constructive exchange between pharmacists and infectious disease specialists, with communication providing opportunities for professional growth. However, organizational issues such as difficulties in reaching prescribers, and hierarchical challenges, often hinder communication.

*“It depends on your experience. Some doctors don’t take kindly to being reminded of things, while others are very approachable and will listen. Some will ignore you just to prove a point”*

(FG 14 nurses) [20]

*“It’s everyone’s responsibility—carers, team leaders, doctors, and nurses—to ensure correct assessments are made when there is a change in condition”*

(Nurse, INT 28) [28]

*Communication between professionals from different care settings*

According to nurses, maintaining the level of communication between every professional belonging to different care settings appears to be a crucial issue in AMS.

*“Good communication between the treating teams and ED (Emergency Department) doctors should be paramount...”*

(P14 Nurse) [29]

*Patient and Caregiver Communication*

Antibiotic prescriptions are often influenced by the expectations of patients and caregivers, rather than clinical necessity.

*“My husband is having dengue; do you have any antibiotics for him?”*

(FGD011, SGH) [6]

*“A man told me, ‘I’m the one who decides,’ but his relatives persuaded him when we weren’t present. Family members often complicate the situation”*

(Nurse) [30]

**Nurses’ Role**

Fifteen findings (15 U) explored nurses’ roles in AMS.

*Prescribing and Decision-Making Processes*

Some nurses recognize their responsibility in ensuring proper antibiotic prescription and administration, while others feel unprepared to participate in antimicrobial decision-making.

*“It’s everyone’s responsibility to check that the correct dose has been given”*

(F11, private theatre nurse) [31]

*Promoter of Professional Dialogue*

Nurses agreed that discussing antimicrobial management and understanding guidelines are essential elements for fostering a culture of change.

*“We have the guidelines online, and we can print them out. Even though we can’t be part of the actual prescribing decision, we can be part of the discussion and raise concerns, which could initiate a culture change”*

(F11, private theatre nurse) [31]

*Advocate for Patients*

Nurses emphasized that effective communication with family members is crucial in advocating for the patient, making it essential to establish a care plan in the patient’s best interest.

*“It’s our responsibility to advocate for the patient. If we can reduce the amount of unnecessary antibiotics, we should. It’s essential that we get involved”*

(nurse manager) [8]

*Leadership and Motivator Roles*

Nurses often take on leadership roles in various settings.

*“In our unit, we spread the word. For UTIs, we don’t just ask for antibiotics; we look for at least three symptoms before deciding. Otherwise, we try other methods like increasing fluids and observing for a few days first”*

(Nurse 9, p. 692) [25]

*Infection control role*

All nurses agreed that infection control is critical in nursing practice and requires a comprehensive approach, including surveillance, support, and education.

*“In the morning, we all check lab results to see if patients have multidrug-resistant infections, especially in the ICU. We also verify that the antibiotics prescribed align with microbiology results”*

(ICN 4) [21]

## 4. Discussion

From the studies analyzed, it is apparent that the level of knowledge, understanding, and acceptance of AMS among nurses is inconsistent. This variability appears to depend primarily on the geographic location in which care is provided [14,21,26,27], as analyzed in our results. While differences across hospital, residential, or community settings were not directly assessed, future research may explore how these structures could influence knowledge and acceptance of AMS among nurses.

Additionally, the lack of awareness about AMS programs, coupled with the difficulty in promoting, sharing, and applying them, is closely linked to the availability of institutional support [24,32], economic resources, antibiotic resistance patterns, and the absence of comprehensive education on the topic.

AMS programs in Europe and North America are supported by national policies specifically designed to combat antimicrobial resistance (AMR) [33,34,35]. However, the situation in Africa is markedly different. In 2017, a World Health Organization (WHO) survey revealed that only two countries (4.3%) on the continent promote national AMS plans, and just 14.9% have national policies addressing infection prevention and control (IPC) [36]. Furthermore, no African country currently utilizes a national AMR surveillance system capable of providing reliable, timely, and comparable data on the appropriateness of antibiotic use. The lack of active research, the limited development of new drugs, and inadequate training and educational strategies further hinder progress in this area [36].

Key aspects of AMS reported by the interviewees include the active role of nurses, with a focus on organizational, contextual, and communication aspects, as well as knowledge, training, and the promotion of programs aimed at improving antibiotic use. The role of healthcare organizations in promoting AMS programs is considered critically important (WHO, 2019). Several studies have emphasized the need for organizational changes not only at the local level but also nationally and internationally. 

Nurses frequently identified high workloads as a significant barrier to AMS implementation. A review on missed nursing care showed that increased workloads are associated with a higher incidence of adverse outcomes, including medication errors [35]. The issue of understaffing is particularly problematic in Italy, where the nurse-to-population ratio (6.2 per 1000 inhabitants) is 25% below the European Union average [37].

Some nurses also reported that compliance with specific accreditation requirements served as a motivating factor for their participation in AMS programs [24]. Additionally, the review of the studies highlighted the lack of material and economic resources as a potential hindrance to AMS compliance and implementation. One of the meta-synthesis studies reported that the absence of an effective computerized system could lead to transcription errors and, consequently, incorrect prescribing by physicians.

A further point emerging from the analysis is the critical importance of nurse education regarding AMS programs. While the need for more in-depth knowledge of AMS was frequently expressed by interviewees, some studies noted that nurses were already participating in AMS-related activities, even though their contributions were neither formally recognized nor acknowledged by the nurses themselves [6,8,14,25]. This reflects an underlying need to formally integrate nurses into AMS initiatives.

The literature consistently underscores the need to change the knowledge, attitudes, and behaviors of healthcare professionals to promote the appropriate use of antibiotics, improve patient outcomes, reduce AMR, and decrease the prevalence of healthcare-associated infections (HAIs) caused by multidrug-resistant organisms (MDROs) [1,7]. In fact, as previously pointed out, a gap in adherence to fundamental hand hygiene practices has been observed, which underscores the need for ongoing professional development and reinforcement of these behaviors. We acknowledge that handwashing is introduced as a fundamental skill during undergraduate training. However, as guidelines and best practices evolve, it is critical that nurses continue to receive training throughout their careers to ensure adherence. This does not imply that postgraduate courses should replace undergraduate instruction on basic hand hygiene; instead, ongoing education at all levels is necessary to reinforce and update these skills, particularly in complex healthcare environments where lapses in compliance may occur.

However, in some settings, HAIs are so prevalent that they no longer provoke concern among nurses, diminishing their motivation to pursue further education, as HAIs are often viewed as unavoidable [14].

In terms of healthcare personnel training, the situation in Italy is currently in flux, particularly following the 2017 country visit by the European Centre for Disease Prevention and Control (ECDC) [38]. The ECDC report highlighted a significant lack of specific training programs related to HAI prevention in both basic and specialist curricula. In alignment with the fundamental principles of the National Plan Against Antimicrobial Resistance (PNCAR), substantial emphasis is now placed on training, as recommended by the WHO’s competency framework for health worker education on antimicrobial resistance (2018) [39].

The Italian Group for the Study of Hospital Hygiene (GISIO) advocates for the implementation of pre- and post-graduate training programs for all healthcare professionals involved in AMS to ensure adequate professional preparation [40]. This necessity was echoed by the nurses interviewed, who stressed the importance of strengthening their role to facilitate workflow and enhance patient safety [6,25]. In this sense, even the obstacles reported by nurses to AMS participation can serve as catalysts for the development of innovative and challenging strategies.

## 5. Conclusions

This meta-synthesis underscores the crucial yet often underappreciated role of nurses in antimicrobial stewardship (AMS) programs. Nurses, due to their direct and sustained contact with patients, are ideally placed to influence antibiotic use and ensure adherence to AMS protocols. However, the findings from this study reveal that their involvement in AMS remains limited by several factors, including organizational barriers, insufficient training, and a lack of recognition for their potential contributions.

From the nurses’ perspective, they are frequently marginalized in AMS discussions, despite their unique position to observe patient outcomes, administer antibiotics, and provide feedback to physicians and pharmacists. According to the nurses, this gap in involvement is partly due to the physician-dominated nature of AMS programs, which tend to overlook the nursing perspective. Expanding the role of nurses within AMS teams can have several positive outcomes, such as more consistent monitoring of antibiotic use, enhanced communication between multidisciplinary teams, and improved patient safety. Empowering nurses with more leadership opportunities in AMS could not only enhance the effectiveness of these programs but also promote a culture of shared responsibility in combating antimicrobial resistance (AMR). 

The primary barriers identified in this meta-synthesis include high workloads, insufficient resources, and inadequate training. Nurses reported that the complexity of their tasks, combined with a shortage of staff and the use of cumbersome documentation systems, often led to shortcuts in antimicrobial practices, potentially undermining the effectiveness of AMS. Additionally, the lack of IT systems that facilitate real-time data sharing and patient monitoring further hinders nurses’ engagement in AMS activities. Addressing these barriers through targeted investment in healthcare infrastructure, streamlined workflows, and adequate staffing will be essential to enhancing nurse participation in AMS. 

Another challenge is the variability in the level of knowledge and understanding of AMS across different settings. In some countries, nurses have access to comprehensive training and guidelines on infection control and antimicrobial practices. However, in other regions, especially low- and middle-income countries, training on AMS is either inadequate or non-existent. This gap calls for a more globalized approach to AMS training, ensuring that nurses in all healthcare settings are equipped with the knowledge and tools needed to effectively participate in antimicrobial stewardship. 

Healthcare policymakers must recognize the vital role nurses can play in the fight against AMR. Formal recognition of their contributions to AMS, along with the development of policies that encourage greater nurse involvement, would be a significant step forward. Such policies might include creating AMS leadership positions for nurses, mandating AMS education in nursing curricula, and promoting interdisciplinary collaboration within AMS teams. Additionally, national and international health organizations should consider establishing standardized AMS protocols that explicitly define the role of nurses in these programs. Further research is needed to explore how nurses can be more effectively integrated into AMS programs. While this study focuses primarily on nurses’ perceptions and experiences, additional studies could examine the impact of nurse-led AMS initiatives on patient outcomes, antibiotic prescribing practices, and overall healthcare system efficiency. It would also be beneficial to investigate the influence of cultural, organizational, and geographical factors on nurses’ involvement in AMS, as the level of engagement appears to vary significantly between different regions and healthcare systems.

In conclusion, while the active involvement of nurses in AMS has been minimized, their potential to contribute meaningfully to tackle AMR is relevant. By addressing the structural, educational, and cultural barriers that limit their participation, healthcare systems might leverage nurses’ skills and insights and improve antibiotic stewardship, patient safety, and healthcare outcomes. A collective approach of healthcare organizations, educators, and policymakers is required to fully integrate nurses into AMS programs, ensuring that they are equipped with the necessary skills to help mitigate the negative impact of AMR. This approach is not only necessary for the success of AMS programs but also critical for safeguarding public health in the face of rising antibiotic resistance.

## Figures and Tables

**Table 1:** Data extraction and synthesis.

**Metasynthesis 1: AMS Programme**	
**Findings**	**Category**
Not participating in the AMS programme (U)	Adherence to programmes
No feedback about AMR in the unit (U)	
AMS efficacy in ICU (U)	
Individual perceptions of the need for AMS (U)	Individual perceptions and nursing participation in the AMS
Frustration and burnout (U)	
Nurses partecipation in AMS (U)	
No perceive AMR as an issue (U)	
Nurses’ role in formal AMS policies not evident (U)	Role of the nurse in the AMS from the perspective of other health professions
Traditional roles exclude nurses (U)	
Nurses’ role should focus on providing patient care (U)	
**Metasynthesis 2: Organization, context and resources**	
**Findings**	**Category**
Taking shortcuts by altering a procedure (U)	Workload
Turnover patient (U)	
Inadequate nursing staffing to provide in-service training (C)	
Scarce resources (U)	Material and economic resources
Administrative work (U)	
Cost is a driving force of AMS and antimicrobial prescribing (U)	
IT systems are not conducive to engagement (U)	
Accessing information, resources and reminders (U)	Mode of transmission of information
Competing priorities (C)	
Lack of staff engagement with the AMS program (C)	
Motivation of ACH nurses: accreditation standards (U)	Accreditation standards
Multidisciplinary teamwork (U)	Promotion of multi-professional working and advanced traininig for nurses
Implementation of APRNs (U)	
Legislative framework (U)	Legislative support
**Metasynthesis 3: Training, knowledge and education**	
**Findings**	**Category**
Nurses are not educationally prepared to engage fully in AMS (U)	Training in university courses
ID and Antibiotic knowledge (U)	
Lack of information/Knowledge (U)	
Not understanding IPC: inadeguate basic nursing knowledge (U)	Postgraduate training
Allowing opportunities for learning (U)	Nurses’ knowledge about antibiotics and the influence on AMS
Varied antimicrobial knowledge and experience (U)	
Inadequate knowledge of the principles of antibiotic use inhibits day-to -day engagement (U)	
AMS Awareness (U)	
Knowledge deficit (U)	
Lack of ongoing formal education in culturing techniques (U)	Guideline training
Nurses with less experienced that following nursing practice protocols (U)	
Antibiotic administration guidelines and departmental practices (U)	
Clinicians value their autonomy (U)	
**Metasynthesis 4: Use of antibiotics**	
**Findings**	**Category**
Antibiotic prescription (U)	Prescribing antibiotics
Task shifting related to antibiotic prescriptions (U)	
Inappropriate prescription (U)	
Optimising antibiotic use (U)	
Overuse of antibiotics (U)	
Excessive and uncontrolled use of antimicrobials in the community for plant and animal farming indirectly promotes antimicrobial resistance (U)	
Unregulated sale of over-the-counter antibiotics (U)	Accessibility to antibiotics
Perceived demands for “quick cures” influenced the use of antibiotics (U)	
Capabilities of ACH nurses: Benchmarking antimicrobial use, medication reviews, and goal setting (U)	Benchmarking
**Metasynthesis 5: Communication and relationship**	
**Findings**	**Category**
Nurses’ confidence with doctor’s decisions (U)	Relationship and communication between different professionals
Collaboration and nurse engagement (U)	
Misalignment of the work and tools of information sharing within the facility (U)	
Difficulty reaching prescribers for direct interaction (U)	
Encouraging bedside nurses in AMS intervention (C)	
Team approach (U)	
Nurses’ interaction with other healthcare professionals (U)	
Infectious disease and antibiotic experts (U)	
Better communication between teams’ communication (U)	Communication between professionals from different care settings
Improved Communication (C)	
Pressure for antibiotic treatment from relatives (U)	Communication and relationship between patients/caregivers and health professionals
Engaging with families (U)	
Nurses’ interaction with patients and their caregivers (U)	
Public awareness and knowledge on antibiotics use and AMR (U)	
**Metasynthesis 6: Nurses’ role**	
**Findings**	**Category**
Nurses’ critical role in prompting post-operative antimicrobial review (U)	Role as guarantor of antimicrobial prescribing and involvement in decision making processes
Nurses’ role in surgical antimicrobial prophylaxis (U)	
Outside the nurses’ scope of practice (U)	
Empowered in the role of antibiotics administration (U)	
Nurses not part of decision-making (U)	Role as guarantor of antimicrobial prescribing and involvement in decision-making processes/ Role as promoter of dialogue between professionals
Nurses’ decision making (U)	Role as promoter of dialogue between professionals
Nursing role in AMS (U)	
Constant presence (U)	Patients’ advocacy role
Motivations of ACH nurses: Resident advocates and following nursing practice protocols (U)	
Motivation of ACH nurses for antimicrobials and for AMS (social/professional role, identify of nurses, beliefs about consequences, emotion): meeting the perceived expectations of family (U)	
Nurse AMS leadership (U)	Role of motivator and leadership
Motivation of ACH nurses: nurses who undertake AMS are optimistic about their capabilities (U)	
Motivations of ACH nurses: goal setting as an AMS motivator for nurses (U)	
Leadership is needed for AMS success (U)	
Infection control nurses (ICNs): role in surveillance, support and education (U)	Infection control role: surveillance, support and education

U, unequivocal, C, credible.

**Table 2:** Data confidence evaluation.

Antimicrobial Stewardship in Healthcare: Exploring the Role of Nurses in Promoting Change, Identifying Barrier Elements and Facilitator—A Metasynthesis					
Synthesized Finding	Type of Research	Dependability	Credibility	ConQual Score	Comments
AMS Programme	Qualitative	High	High	High	Dependability: all studies scored high dependability. Credibility: given the results obtained (10 U) a high level of credibility was declared.
Organisation, context, and resources	Qualitative	High	Moderate (Downgraded one level)	Moderate	Dependability: all studies scored high dependability. Credibility: Given the results obtained, it was downgraded by one level (11 U—3 C).
Training, knowledge, and education	Qualitative	High	High	High	Dependability: all studies scored high dependability. Credibility: given the results obtained (13 U) a high level of credibility was declared
Antibiotic use	Qualitative	Moderate (Downgraded one level)	High	Moderate	Dependability: Five studies scored high reliability and 2 scored moderate reliability Credibility: given the results obtained (9 U) a high level of credibility was declared
Communication and relationship	Qualitative	High	Moderate (Downgraded one level)	Moderate	Dependability: all studies scored high dependability
					Credibility: Given the results obtained, it was downgraded by one level (12 U—2 C results
Nurses’ role	Qualitative	Moderate (Downgraded one level)	High	Moderate	Dependability: Seven studies scored high reliability and 1 scored moderate reliability Credibility: given the results obtained (15 U) a high level of credibility was declared

**Table 3:** Summary of meta-synthesis.

Metasynthesis	Findings	Categories
AMS Program	10	3
Organization, context and resources	14	6
Training, knowledge and education	13	4
Use of antibiotics	9	3
Communication and Relationship	14	3
Nurses’ role	15	5
Total metasynthesis = 6	Total findings = 75	Total categories = 24

## Data Availability

The original contributions presented in the study are included in the article/Supplementary Materials, further inquiries can be directed to the corresponding author.

## Supplementary Materials

The following supporting information can be downloaded at: https://www.mdpi.com/article/10.3390/healthcare12212122/s1, Table S1: Data extraction and synthesis.

## References

[1] Antimicrobial Stewardship Programmes in Health-Care Facilities in Low- and Middle-Income Countries. https://www.who.int/publications-detail-redirect/9789241515481.

[2] Stemming the Superbug Tide: Just a Few Dollars More | en | OECD. https://www.oecd.org/health/stemming-the-superbug-tide-9789264307599-en.htm.

[3] Cassini A., Högberg L.D., Plachouras D., Quattrocchi A., Hoxha A., Simonsen G.S., Colomb-Cotinat M., Kretzschmar M.E., Devleesschauwer B., Cecchini M. (2019). Attributable deaths and disability-adjusted life-years caused by infections with antibiotic-resistant bacteria in the EU and the European Economic Area in 2015: A population-level modelling analysis. Lancet Infect Dis..

[4] Ministero della Salute Piano Nazionale Contrasto Antibiotico-Resistenza—PNCAR. https://www.salute.gov.it/portale/antibioticoresistenza/dettaglioContenutiAntibioticoResistenza.jsp?id=5281&area=antibiotico-resistenza&menu=vuoto.

[5] Antimicrobial Stewardship—From Principles to Practice e-book. https://bsac.org.uk/antimicrobial-stewardship-from-principles-to-practice-e-book/.

[6] Wong L.H., Bin Ibrahim M.A., Guo H., Kwa A.L., Lum L.H., Ng T.M., Chung J.S., Somani J., Lye D.C., Chow A. (2020). Empowerment of nurses in antibiotic stewardship: A social ecological qualitative analysis. J. Hosp. Infect..

[7] Olans R.D., Olans R.N., Witt D.J. (2017). Good Nursing Is Good Antibiotic Stewardship. Am. J. Nurs..

[8] Carter E.J., Greendyke W.G., Furuya E.Y., Srinivasan A., Shelley A.N., Bothra A., Saiman L., Larson E.L. (2018). Exploring the nurses’ role in antibiotic stewardship: A multisite qualitative study of nurses and infection preventionists. Am. J. Infect. Control..

[9] Anwar M., Raziq A., Shoaib M., Baloch N.S., Raza S., Sajjad B., Sadaf N., Iqbal Z., Ishaq R., Haider S. (2021). Exploring Nurses’ Perception of Antibiotic Use and Resistance: A Qualitative Inquiry. J. Multidiscip. Healthc..

[10] Lockwood C., Porritt K., Munn Z., Rittenmeyer L., Salmond S., Bjerrum M., Loveday H., Carrier J., Stannard D. (2024). Chapter 2: Systematic reviews of qualitative evidence. JBI Manual for Evidence Synthesis.

[11] Cooke A., Smith D., Booth A. (2012). Beyond PICO: The SPIDER Tool for Qualitative Evidence Synthesis. Qual. Health Res..

[12] Page M.J., McKenzie J.E., Bossuyt P.M., Boutron I., Hoffmann T.C., Mulrow C.D., Shamseer L., Tetzlaff J.M., Akl E.A., Brennan S.E. (2021). The PRISMA 2020 statement: An updated guideline for reporting systematic reviews. BMJ.

[13] Critical Appraisal Skills Programme (2018). CASP Qualitative Studies Checklist. https://casp-uk.net/casp-tools-checklists/qualitative-studies-checklist/.

[14] Ayton D., Watson E., Betts J.M., Doyle J., Teh B., Valoppi G., Cotta M., Robertson M., Peel T. (2022). Implementation of an antimicrobial stewardship program in the Australian private hospital system: Qualitative study of attitudes to antimicrobial resistance and antimicrobial stewardship. BMC Health Serv. Res..

[15] Rout J., Brysiewicz P. (2020). Perceived barriers to the development of the antimicrobial stewardship role of the nurse in intensive care: Views of healthcare professionals. S. Afr. J. Crit. Care.

[16] Ramly E., Tong M., Bondar S., Ford J.H., Nace D.A., Crnich C.J. (2020). Workflow Barriers and Strategies to Reduce Antibiotic Overuse in Nursing Homes. J. Am. Geriatr. Soc..

[17] Tadzong-Awasum G. (2023). Health personnel experiences with antimicrobial resistance: A qualitative phenomenological assessment in a low-resource setting. Int. Public. Health J..

[18] Munn Z., Porritt K., Lockwood C., Aromataris E., Pearson A. (2014). Establishing confidence in the output of qualitative research synthesis: The ConQual approach. BMC Med. Res. Methodol..

[19] Turner R., Hart J., Ashiru-Oredope D., Atkins L., Eades C., Felton T., Howlett E., Rice S., Shallcross L., Lorencatto F. (2023). A qualitative interview study applying the COM-B model to explore how hospital-based trainers implement antimicrobial stewardship education and training in UK hospital-based care. BMC Health Serv. Res..

[20] Currie K., Laidlaw R., Ness V., Gozdzielewska L., Malcom W., Sneddon J., Seaton R.A., Flowers P. (2020). Mechanisms affecting the implementation of a national antimicrobial stewardship programme; multi-professional perspectives explained using normalisation process theory. Antimicrob. Resist. Infect. Control.

[21] Van Gulik N., Hutchinson A., Considine J., Driscoll A., Malathum K., Botti M. (2021). Perceived roles and barriers to nurses’ engagement in antimicrobial stewardship: A Thai qualitative case study. Infect. Dis. Health.

[22] Mula C.T., Human N., Middleton L. (2019). An exploration of workarounds and their perceived impact on antibiotic stewardship in the adult medical wards of a referral hospital in Malawi: A qualitative study. BMC Health Serv. Res..

[23] Groumoutis J.Y., Gorman S.K., Beach J.E. (2023). Identifying opportunities for antimicrobial stewardship in a tertiary intensive care unit: A qualitative study. Can. J. Crit. Care Nurs..

[24] Jeffs L., McIsaac W., Zahradnik M., Senthinathan A., Dresser L., McIntyre M., Tannenbaum D., Bell C., Morris A. (2020). Barriers and facilitators to the uptake of an antimicrobial stewardship program in primary care: A qualitative study. PLoS ONE.

[25] Dowson L., Friedman N.D., Marshall C., Stuart R.L., Buising K., Rajkhowa A., Gotterson F., Kong D.C. (2020). Antimicrobial stewardship near the end of life in aged care homes. Am. J. Infect. Control..

[26] Bridey C., Le Dref G., Bocquier A., Bonnay S., Pulcini C., Thilly N. (2022). Nurses’ perceptions of the potential evolution of their role in antibiotic stewardship in nursing homes: A French qualitative study. JAC-Antimicrob. Resist..

[27] Nair M., Tripathi S., Mazumdar S., Mahajan R., Harshana A., Pereira A., Jimenez C., Halder D., Burza S. (2019). “Without antibiotics, I cannot treat”: A qualitative study of antibiotic use in Paschim Bardhaman district of West Bengal, India. PLoS ONE.

[28] Hall J., Hawkins O., Montgomery A., Singh S., Mullan J., Degeling C. (2022). Dismantling antibiotic infrastructures in residential aged care: The invisible work of antimicrobial stewardship (AMS). Soc. Sci. Med..

[29] Goulopoulos A., Rofe O., Kong D., Maclean A., O’Reilly M. (2019). Attitudes and beliefs of Australian emergency department clinicians on antimicrobial stewardship in the emergency department: A qualitative study. Emerg. Med. Australas. EMA.

[30] Harbin N.J., Lindbæk M., Romøren M. (2022). Barriers and facilitators of appropriate antibiotic use in primary care institutions after an antibiotic quality improvement program—A nested qualitative study. BMC Geriatr..

[31] Ierano C., Rajkhowa A., Gotterson F., Marshall C., Peel T., Ayton D., Thursky K. (2022). Opportunities for nurse involvement in surgical antimicrobial stewardship strategies: A qualitative study. Int. J. Nurs. Stud..

[32] Ierano C., Thursky K., Peel T., Rajkhowa A., Marshall C., Ayton D. (2019). Influences on surgical antimicrobial prophylaxis decision making by surgical craft groups, anaesthetists, pharmacists and nurses in public and private hospitals. Gurgel RQ, ed. PLoS ONE.

[33] GOV.UK UK 20-Year Vision for Antimicrobial Resistance. https://www.gov.uk/government/publications/uk-20-year-vision-for-antimicrobial-resistance.

[34] WHO Regional Office for Europe, European Centre for Disease Prevention and Control (2023). Surveillance of Antimicrobial Resistance in Europe, 2022 Data: Executive Summary.

[35] Jones L.F., Hawking M.K.D., Owens R., Lecky D., Francis N.A., Butler C., Gal M., McNulty C.A.M. (2018). An evaluation of the TARGET (Treat Antibiotics Responsibly; Guidance, Education, Tools) Antibiotics Toolkit to improve antimicrobial stewardship in primary care-is it fit for purpose?. Fam. Pract..

[36] Essack S.Y., Desta A.T., Abotsi R.E., Agoba E.E. (2017). Antimicrobial resistance in the WHO African region: Current status and roadmap for action. J. Public. Health.

[37] OCSE/Osservatorio Europeo Delle Politiche e Dei Sistemi Sanitari (2021). Italia: Profilo Della Sanità 2021.

[38] European Centre for Disease Prevention and Control (2017). ECDC Country Visit to Italy to Discuss Antimicrobial Resistance Issues.

[39] WHO (2018). Competency Framework for Health Workers’ Education and Training on Antimicrobial Resistance.

[40] Agodi A., Auxilia F., Brusaferro S., Chiesa R., D’Alessandro D., D’Errico M.M., Finzi G., Meledandri M., Mongardi M., Montagna M.T. (2014). Education and training in patient safety and prevention and control of healthcare associated infections. Epidemiol. Prev..

