# Supplementary material for: Antimicrobial Stewardship in Healthcare: Exploring the Role of Nurses in Promoting Change, Identifying Barrier Elements and Facilitators—A Meta-Synthesis
Source: Healthcare (Basel). 2024 Oct 24;12(21):2122. doi: 10.3390/healthcare12212122 (PMC11544781; doi:10.3390/healthcare12212122)
Supplement: Supplementary file 1 [file healthcare-12-02122-s001.zip › healthcare-3236139-supplementary.pdf]

Supplementary Materials

Title	Author/ Year/ country	Method For Data Collection And Analysis	Phenomens Of Interest	Setting/Context/Culture	Partecipant Characteristics And Sample Size	Description Of Main Results
A qualitative interview study applying the COM-B model to explore how hospital-based trainers implement antimicrobial stewardship education and training in UK hospital-based care (ART. 1 PUBMED)	Turner et al 2023 United Kingdom	Qualitative study with semi-structured virtual interviews, analysed using inductive thematic analysis, followed by deductive analysis using the COM-B model, guided by criteria for reporting qualitative research COREQ.	Explore the factors influencing the implementation of AMS E&T (education and training).	UK hospitals	34 participants: 3 nurses, 26 antimicrobial pharmacists, 1 advanced clinical practioner, 2 infectious disease consultants, 1 microbiologist and 1 clinical scientist. They were responsible, as part of their job role, implementation, and evaluation of AMS E&T.	This study has identified several factors that influenced implementation of AMS E&T in UK hospital (wider contextual issues, inter-professional relationships, and individual beliefs) and further identified where implemented, AMS E&T did not address real-world challenges. AMS E&T needs to be optimised to elict practise change, with recommendations including training and engaging the wider work-force.
Mechanisms affecting the implementation of a national	Currie et al. 2020 United Kingdom	Exploratory qualitative study used in-depth telephone	Explaine mechanisms affecting the implementatio	Acute-care hospitals across Scotland	27 total AMT participants across 14 NHS Health Boards: 9 AMT Lead	The beneficial impact of a national approach towards AMS,

antimicrobial stewardship programme; multi-professional perspectives explained using normalisation process theory (ART. 2 PUBMED)		interviews and focus group. Data was first thematically analysed, barriers and enablers were then categorised and Normalisation Process Theory (NPT) was used.	n of a national antimicrobial stewardship programme from multi-professional perspectives.		infection specialist consultant, 15 AMT pharmacists and 3 AMT nurses.<br>72 total front-line practitioner participants across 5 NHS Health Boards (15 focus group): 21 prescribing doctors, 28 clinical pharmacists and 23 ward based nurses<br>Total study participants: 99	with strategic leadership rolled out at local level. It indicated that major barriers relate to organisational context and resource availability. AMS was coherent with implementation leads and prescribing doctors, less for consultants and nurses who may not access training.<br>Important implications internationally for others seek to implement AMS in the hospital setting. AMS interventions can have beneficial impact on appropriate antibiotic prescribing and

						reductions in antibiotic resistant infections.
Opportunities for nurse involvement in surgical antimicrobial stewardship strategies: A qualitative study (ART. 3 PUBMED)	Ierano et al. 2020 Australia.	An exploratory multi-site collective qualitative study with transcribed audio-recording, thematic analisys and with mapping to established frameworks. This secondary study builds upon the existing qualitative research.	Aims to provide greater understanding of the nurses' role in the surgical setting and now to support their practise-relevant engagement in surgical antimicrobial stewardship.	Surgical setting. Melbourne, Australia	Purposive and snowball sampling strategies were implemented to recruit surgeons, anaesthetists, pharmacists and theatre nurses across 3 hospitals in Melbourne. Fourteen focus-group and one paired interviews were completed. Two of them included nurses. Focus group 1 (F1) consisted of 8 public theatre nurses and F11 involved 5 private theatre nurses.	Many opportunities exist for nurse engagement in surgical antimicrobial stewardship. Identification of barriers and enablers support theoretically informed strategies, education and guideline accessibility, multidisciplinary collaborations, executive support for nursing capacity building and the standardisation of surgical antimicrobial prophylaxis workflow and documentation.

						Nurses are critical to patient safety and to support antimicrobial stewardship.
4 "Without antibiotics, I cannot treat": A qualitative study of antibiotic use in Paschim Bardhaman district of West Bengal, India	Mohit Nair, Santanu Tripathi, Sumit Mazumdar, Raman Mahajan, Amit Harshana, Alan Pereira, Carolina Jimenez, Debasish Halder, Sakib Burza. India 2019	Qualitative study with semi-structured interviews, Qualitative data was analyzed using the framework method in an inductive and deductive manner.	To understand knowledge of appropriate antibiotics and perceptions of antibiotic use among formal and informal healthcare providers,	This study took place in Paschim Bardhaman district of West Bengal, which consists of an urban city centre and eight administrative blocks.	The participants: 6 allopathic doctors, 5 pharmacy shopkeepers, 5 IHPs, 5 nurses and 7 patients accessing care at the OPD setting	This study highlights major shortcomings of the public health system. In some care points there is no doctor, and the nurse feels entitled to prescribe drugs including antibiotics based solely on personal experience (lack of knowledge on the part of the nurses).
5 **Workflow Barriers and Strategies to Reduce Antibiotic Overuse in Nursing Homes**	Edmond Ramly PhD, Michelle Tong MS, Svetlana Bondar MPH, James H. Ford II PhD, David A. Nace MD, MPH, Christoph	Qualitative descriptive study using prospective field-based assessment of workflows.	By analyzing the preprescribing workflow across NHS, researchers try to identify barriers to reducing antibiotic	SETTING: Six NHs in Wisconsin (n = 3) and Pennsylvania (n = 3)	PARTICIPANTS: A total of 44 interviews with 68 NH professionals, including leadership, nurses, and prescribers. 19 providers, 25 nursing staff (80% registered nurses), and 24 leadership staff .	Thematic analysis identified common step-specific barriers to antibiotic stewardship in three themes: (A) information barriers, comprising (A1)

	er J. Crnich MD, PhD.<br>2020 American Geriatrics Society		overuse and strategies to address them to improve antibiotic prescribing.			inconsistent nurse assessment reporting and (A2) misalignment between the work and tools of information sharing within the facility, (B) communication barriers, comprising (B1) mismatched perception of prescriber information needs and (B2) difficulty reaching prescribers for direct interaction, and (C) professional barriers, comprising (C1) low prescriber confidence in nurse assessment report and (C2) nurse reluctance to express their

						professional opinion
6 **Exploring the nurses' role in antibiotic stewardship: A multisite qualitative study of nurses and infection preventionists**	Eileen J. Carter, William G. Greendyke, E. Yoko Furuya, Arjun Srinivasan, Alexa N. Shelley, Aditi Bothra, Lisa Saiman, and Elaine L. Larson	Qualitative study with semi-structured interviews, and focus group with clinical nurses, nurse managers, and IPs who worked in general intensive care units (ICUs) and medical-surgical units of 2 academic hospitals that provide care to adult or paediatric populations in New York City. Data were coded by 3 members of the research team (E.C., A.S., and A.B.) using a conventional content analysis in which data were grouped	The purpose of this descriptive qualitative study was to explore the attitudes of nurses and infection preventionists (IPs) toward 5 of the nurse-driven antibiotic stewardship activities recommended by the ANA/CDC working group. Specifically, that nurses may play a major role in optimizing antibiotic treatment by: 1) questioning the medical necessity of	Setting two hospital NY city	49 clinical nurses, 5 nurse managers, and 7 IPs. All IPs had worked as clinical nurses.	This study highlights how important the role of the nurse could be in antimicrobial stewardship programs, taking into consideration 5 aspects:<br>1 question the need for urine cultures<br>2 ensure the proper cultivation technique<br>3 Record an accurate history of antibiotic allergy<br>4 encourage rapid transition to oral therapy<br>5 initiate an antibiotic timeout.<br>Numerous barriers emerge from the results:<br>1 Lack of knowledge

		according to codes derived from transcripts in NVivo software	urine cultures; 2) ensuring proper urine and blood culturing techniques; 3) initiating the switch from intravenous (IV) to oral (PO) antibiotics; 4) obtaining and recording an accurate penicillin drug allergy history; and 5) initiating an antibiotic timeout.			2 discomfort in questioning doctor's orders<br>3 lack of responsibility regarding appropriate techniques<br>4 lack of awareness of the negative consequences resulting from an inadequate cultivation technique<br>5 difficulties some nurses have in taking on more responsibilities
7 **An exploration of workarounds and their perceived impact on antibiotic stewardship in the adult medical wards of a referral hospital in Malawi: a qualitative study**	Chimwemwe Tusekile Mula, Nicola Human & Lyn Middleton<br>2019 South Africa	This was a qualitative descriptive case study design and is part of a large mixed methods study. This study was conducted with staff interviews observations of	this study explains how nurses try to overcome the obstacles they encounter in healthcare activities, using alternative solutions	a tertiary hospital in Malawi	20 participants including doctors, pharmacists and laboratory technicians divided into 3 focus groups. And 13 nurses.	The study provided insight into how nurses and doctors work around workflow blocks encountered during patient antibiotic management at a

		nurses' antibiotic stewardship practices on two adult medical wards. For the interviews, the focus group technique was used, composed of doctors, pharmacists and laboratory technicians. only at a later stage, 13 nurses were sampled and interviewed				tertiary hospital in Malawi. Two categories of alternative solutions have been identified: 1 take shortcuts by modifying the procedure 2 use unauthorized process steps.
8**Attitudes and beliefs of Australian emergency department clinicians on antimicrobial stewardship In the emergency department:qualitative study** (art 8)	Anne Goulopoulos, Olivia Rofe, David Kong, Andrew Maclea And Mary O'reilly	Semi-structured, one-to-one interviews of a duration of 20–30 min were conducted using profession specific questionnaire guides. Literature was used to develop	The Objective of this study was To explore the attitudes and beliefs of Australian ED clinicians towards antimicrobial stewardship in the ED.	Emergency department clinicians. Australia	Participated: (Twenty-two clinicians: eight doctors, eight nurses and six pharmacists) and hospital administrators working in Australian public hospital EDs	All participants agreed that AMR was a worldwide problem attributed to inappropriate and over-prescribing of antimicrobials and resistance driven from overseas Perceived barriers

		guides that were tested and refined. Interviews were audio-recorded and transcribed			participated,providing source triangulation	to AMS in EDs were considerable and generally related to individual healthcare providers and resourcesFacilitators to judicious antimicrobial use focused on resources, timely and reliable access to expert opinion and knowledge through education to empowerclinician intervention and promote advocacy from within.
**9Barriers and facilitators of appropriate antibiotic use in primary care institutionsafter an**	Nicolay Jonassen Harbin1*, Morten Lindbæk1 and Maria Romøren2	semi-structured<br><br>focus group interviews. used a semi-structured interview guide	Identifying barriers and facilitators of appropriate antibiotic prescribing	NHs and municipal acute care units . Norvegia	physicians (*n* = 11) and nurses (*n* = 14) in 10 NHs and 3 MACUs. The participants did not work in the same	identified thirteen themes grouped into four main overarching levels affecting antibiotic use during the

**antibiotic quality improvementprogram – a nested qualitative stu**dy		covering multiple areas influencing antibiotic used a semi-structured interview guide covering multiple areas influencing antibiotic			institution, except from one interview where one physician and three nurses were employed at the same MACU ward	analysis. Barriers and facilitators at the clinical level, at the resident level, at the next of kin level, and at the organisational level . main finding was the unclear clinical presentation of symptoms and lack of diagnostic possibilities as persistent barriers of appropriate antibiotic use after thequality improvement programme. Increased knowledge and awareness, appropriate use
**10 Barriers and facilitators to the uptake of an antimicrobial stewardship program in primary**	Lianne Jeffs ,Warren McIsaac, Michelle Zahra Linda Dresser6, Mark	Qualitative interviews were conducted Aninterview guide was developed to evaluate	explore the perceptions of primary care prescribers of the usefulness, feasibility, and	interprofessional urban primary care clinics in Toronto, Canada	19 family physicians, 3 pharmacists, and 1 nurse practitioner	identified additional factors with the potential to influence the incorporation of multi-faceted ASP activities into

**care: A qualitative study**	McIntyre6, David Tannenbaum, Chaim Bell,Andrew Morris	participants' perceptions and experiences. The average length of interviews was 16.5 minutes with a range of 11–32 minutes. An iterative conventional content analyses approach. reviewing the transcripts line-by-line separately to identify sections of text that served as codes.	experiences associated with the implementatio n of a pilot community-focused ASP intervention in three primary care clinics			clinical practice. These included having a local champion to handle the organizational issues and ongoing communication, prompts during clinical activities to remind them to think about their ASP and options, having enough time to learn about new ASP information, and ongoing audit and feedback regarding antimicrobial prescribing practices.
11**Empowerment of nurses in antibiotic stewardship: a social ecological qualitative analysis**	L.H. Wong a, M.A. Bin Ibrahim a,k , H. Guo a,b , A.L.H. Kwa c,d , L.H.W. Lum e,f , T.M. Ng g, J.S.	Focus group discussions (FGDs) were conducted with purposively sampled nurses. FGDs were	Understand the facilitators and barriers that impact nurses' involvement and	Three major tertiary-care public hospitals in **Singapore**: 1200-bed National University Hospital (NUH), 1800-bed Singapore General	15 FGDs involving 104 nurses were conducted. Three quarters of the participants had received tertiary education (degree,	**At the intrapersonal level**, nurses felt empowered in carrying out their roles in antibiotic administration.

	Chung f,h , J. Somani e, D.C.B. Lye b,f,i,j , A. Chow a,j,<br><br>2020<br><br>Singapore	audio-recorded and transcribed verbatim. Data were analysed using Applied Thematic Analysis and interpreted using the Social Ecological Model.	empowerment in antibiotic stewardship.	Hospital (SGH), and 1600-bed Tan Tock Seng Hospital (TTSH)	masters, or doctorate) and half had practised in their respective hospitals for 10 years	They saw themselves as gatekeepers to ensure that the prescribed antibiotics were administered appropriately. However, nurses felt they lacked the knowledge and expertise in antibiotic use and AMR prevention. At the interpersonal level, this deficit in knowledge and expertise in antibiotic use impacted how they were perceived by patients and caregivers as well as their interactions with the primary care team when voicing outpatient safety concerns and antibiotic

						administration suggestions. **At the organizational level**, nurses relied on drug administration guidelines to ensure appropriate antibiotic administration and as a safety net when physicians questioned their clinical practice. **At the community level,** nurses felt there was a lack of awareness and knowledge on antibiotic use among the general population.
12 **Implementation of an antimicrobial stewardship program in the Australian private hospital system:**	Darshini Ayton, Eliza Watson, Juliana M. Betts, Joseph Doyle, Benjamin The, Glenn Valoppi,	A mixed-methods study was performed, involving three focus groups with	Explore capabilities, opportunities and motivations for AMR and AMS	Single-centre mixed methods study conducted at a 766-bed private hospital in **Melbourne, Australia**	There were 100 responses to the survey in total, including 32 physicians, 15 surgeons, 19	Staf felt more thorough feedback and monitoring could improve prescribing

**qualitative study of attitudes to antimicrobial resistance and antimicrobial stewardship**	Menino Cotta, Megan Robertson and Trisha Peel<br><br>2022<br><br>Australia	stakeholders. All doctors, nurses and pharmacists at the hospital were invited to complete a survey on their experiences with and awareness of AMR, AMS and antimicrobial prescribing. Survey and focus group data were analysed separately with a process of triangulation applied at the interpretation stage of the analysis to determine whether the findings were convergent, complementary or contradictory	with stakeholders at an Australian private hospital, and identify barriers and enablers 5 years post-implementation of an AMS program comparing with pre-implementation fndings		anaesthetists, 21 nurses and 13 pharmacists.	behaviour but acknowledged difficulty in private hospitals in changing habits of staff who valued autonomy in making prescribing decisions. Half of respondents felt the current AMS restrictions should continue. Executive engagement may be needed to drive system changes across a complex network.

13 **Perceived roles and barriers to nurses' engagement in antimicrobial stewardship: A Thai qualitative case study**	Nantanit van Gulik, Ana Hutchinson, Julie Considine, Andrea Driscoll, Kumthorn Malathum, Mari Botti<br><br>2021<br><br>Thailand	A qualitative descriptive study using thematic analysis approach was conducted. Individual semi structured interviews with organisational leaders and focus groups with infection control and ward nurses were conducted. Guidelines for reporting of a qualitative study (Standards for Reporting Qualitative Research [SRQR]) were used.<br>A combination of semi-structured interviews (with organisational	Explore how organisational multidisciplinary leaders and clinical nurses perceive nurses' roles in AMS in a single organisational site case study based in Thailand, within the current governance, educational and practice context, and the barriers to nurses' engagement in AMS	The setting for this study was a 1000- bed university public hospital located in **Bangkok, Thailand.** The study hospital provides advanced medical services with approximately 5000 outpatient visits per day and over 45,000 in-patient admissions per year	The combined number of organisational leaders and nurses was 33. The 15 organisational leaders interviewed were: health service Director, Director of Nursing, Director of Pharmacy, Chair of the Infection Prevention and Control (IPC) committee, IPC nurse manager, infection control specialists (n Z 2), surgeons (n Z 2), an infectious diseases (ID) physician, operating suite nurse manager, intensive care unit (ICU) nurse manager, AMS and clinical pharmacists (n Z 2) and the Virology Department head scientist. The three nurse focus groups	The key roles nurses currently play in AMS were identified as supporting system processes, monitoring for patient safety, monitoring for optimal antibiotic use and through patient education, however these activities are informal and not well-defined, nor articulated in hospital policy and procedures. A lack of clear articulation of nurses' role and traditional professional hierarchies limits active participation. Inconsistent engagement was perceived as due to a failure to prioritise AMS

		leaders) and focus groups (ICNs and clinical nurses) were used to explore concepts related to the roles of nurses in AMS and the perceived barriers to nurses' engagement in AMS at the study hospital.			comprised: infection control nurses (ICN, n Z 7), senior (>10-years' experience) ward nurses (SRN, n Z 5) and junior (<5-years' experience) ward nurses (JRN, n Z 6).	activities, a lack of formal policies and a need for further education
Antimicrobial stewardship near the end of life in aged care homes.<br>(Art 14 Pubmed)	Leslie Dowson MBioethics, N. Deborah Friedman MD, MPH, MBBS, Caroline Marshall PhD, Grad Dip Clin Epi, MBBS, Rhonda L. Stuart PhD, FRACP, MBBS, Kirsty Buising MD, MPH, MBBS, Arjun Rajkhowa PhD,	Qualitative semi-structured interviews (totally 20 to ensure thematic saturation) coded for understanding behaviour change. Data analysis was developed through a more in-depth process of identifying trends and	The objective of this study was to understand how aged care home health professionals perceive antimicrobial use near the end of life and how they perceive potential antimicrobial	Victoria, Australia in aged care homes. They recognize the importance of AMS programs; these will be implemented.	Health professionals (2 pharmacists, 5 GPs, and 12 nurses, 1 GP registrar) responsible for routine care of residents in ACHs were recruited. Clinicians with specialty knowledge that might skew a study of routine care in ACHs, such as specialty palliative	Two major interpretive themes emerged: AMS activities near the EoL in ACHs need to enable ACH nurses to make decisions substantiated by evidence-based clinical knowledge, and AMS activities near the EoL in ACHs must

	Fiona Gotterson MACN, MN, RN,<br>David C.M. Kong PhD, MPharm, BPharm.<br>2019<br>Australia	patterns in the data: The TDF and the COM-B framework. Finally, has been used the recommendatio ns of Fleming et al, to focus the direction of analysis on capabilities and motivations.	stewardship activities<br>near the end of life in aged care homes.		care clinicians, were deliberately not included.<br>Participants were recruited using purposive, convenience, and snowball sampling until data saturation was reached, which was further verified through additional interviews with each profession to ensure<br>that no new themes emerged.<br>Participants chose the setting of<br>their interview and were interviewed once.	address family confidence in resident wellbeing.
Perceived barriers to the development of the antimicrobial stewardship role of the nurse in intensive care: Views of<br>healthcare professionals	J Rout, MN; P Brysiewicz, PhD<br>2020<br>South Africa	This is part of a larger study exploring the role of the ICU nurse in AMS. Using a qualitative research approach, purposive	To explore the views of healthcare professionals regarding barriers to the antimicrobial stewardship role of the	Private hospital in KwaZulu-Natal, South Africa.<br>Nurse intensive care.<br>AMS is now accepted practice in hospitals, particularly within high-risk areas such as the intensive care unit.	Nursing participants included two nurses from hospital management who had set up the AMS programme two years previously, and six clinical ICU	The following categories and subcategories were derived: regarding barriers to the role of the nurse in antimicrobial stewardship: (i) lack

(Art 15 Google Scholar)		sampling was used to identify fifteen participants in a general intensive care unit. Individual semi-structured individual interviews with all participants were held in a private room in the hospital during on-duty time. All interviews were conducted by the principal investigator (JR) who worked in the unit as a clinical nurse. Interviews were transcribed verbatim and were read and reread in a process of submersion. Manifest	nurse in intensive care.		nurses whose responsibilities as shift leaders included daily AMS rounds. These were registered professional nurses; one held an additional qualification as an ICU nurse, the remaining five had experience of working in this area of nursing but held no specialist qualification. Non-nursing participants included a microbiologist, pharmacist, two anaesthetists, two physicians and two surgeons.	of collaboration (subcategories: not participating in the antimicrobial stewardship programme, no feedback about antimicrobial resistance in the unit, and not part of decision-making); (ii) inadequate knowledge (subcategories: not understanding infection prevention and control, missing the link between laboratory results and start of treatment, and poor knowledge of antibiotics and their administration); and (iii) inexperienced

		content analysis was used to identify categories arising from the data.				nurses (subcategories: shortage of intensive care nurses, lack of experienced nurses, and inadequate nursing staff to provide in-service training).
Dismantling antibiotic infrastructures in residential aged care: The invisible work of antimicrobial stewardship (AMS)<br><br>(Art 17 Embase)	Julie Hall, Olivia Hawkins, Amy Montgomery, Saniya Singh, Judy Mullan, Chris Degeling 2022 Australia	This qualitative interview-based study draws on the experiences and perspectives of RACF. Interviews were concluded at the point of data saturation of data redundancy after 56 interviews were completed, based on ongoing review and comparison of interview data	Attention to less visible aspects of antimicrobial stewardship (AMS) in residential aged care and their implications for nurse-led optimization of antibiotic use in these settings. Develope an account of the perceptions, experiences and	Australian residential aged care facilities (RACF).<br>Efforts towards enhancing AMS in RACFs are ongoing. AMS is governed by aged care quality standards that require aged care providers to develop and implement a clinical governance framework.	The sample of 56 participants representing all the key roles in RACFs with a broad range of levels of experience.<br>-10 Managers<br>-19 Nursing staff di cui 16 RNs (infermieri registrati con responsabilità di dirigere e supervisionare) e 3 ENs (infermieri iscritti)<br>-14 Personal care team leader<br>-13 Personal care assistants (PCAs)	This article reports on selected findings relating to how RACF staff perceive, experience and practice in ways that have impacts on AMS. Reflecting the focus of the inductive and deductive codes the key themes that emerged from the analysis related to perceptions and practices

		for emergent themes. Some interview questions were refined, and another 36 interviews were conducted to further focus on emerging key themes of interest. Following the precepts of Framework methodologies, all of this information was entered into a separate tabular matrix to systematically summarise coded data for inductive synthesis and aide deductive contrast and comparison.	practices of staff regarding the 'on the ground' work associated with implementing and upholding AMS objectives and extend research on attempts to dismantle antibiotic infrastructures.			surrounding: (i) antibiotic use; (ii) uncertainties and risk of infection management; (iii) behind the scenes work in AMS practice (negotiation, care and administrative work); and (iv) the importance of a team approach.

Health personnel experiences with antimicrobial resistance: A qualitative phenomenological assessment in a low-resource setting<br>(art 18 embase)	Tadzong-awasum et al. 2022<br>Cameroon<br><br>(Grace Tadzong-Awasum, PhD, MSN, MPH, PG Cert IC, CGNC. Cameroon 2022)	A qualitative phenomenological study was used on a sample of seven health personnel; content analysis of data consisted of bringing similar information together in larger groups and later coded into simpler group.	The objective of this study is to understand health personnel point of view and experiences regarding antimicrobial resistance.	Hospital in Younde, Cameroon.	Seven Health personnel (two certified physicians and five registered nurses) who prescribe and administer antimicrobial on a daily basis in Yaounde, Cameroon.	After data analysis, the following themes were identified as the main themes of the study: 1) overuse of antibiotics following over prescription is a major factor in antibiotic resistance, 2) unregulated sale of over-the-counter antibiotics makes it difficult to manage, 3) excessive and uncontrolled use of antimicrobials in animal and plant breeding promotes resistance, 4) inadequate surveillance and follow-up measures.

Identifying opportunities for antimicrobial stewardship in a tertiary intensive care unit: A qualitative study<br>(19 Embase)	Groumoutis et al. 2023<br>Canada<br><br>(John Y. Groumoutis, BSc (Pharma), ACPR, Sean K. Gorman, BSc (Pharm), ACPR, PharmD, and Jessica E. Beach, BSc. Pharm, ACPR, PharmD. Canada<br>2023)	Semi-structured, individual, in-person, audio-recorded interviews lasting 20-30 minutes were conducted in the hospital; and a thematic analysis was then performed to identify and classify emerging themes.	Understanding the priorities and preferences of intensive care unit health care personnel, identifying opportunities for antimicrobial stewardship and possible AMS interventions to be implemented in the ICU	Hospital's intensive care unit in British Columbia, Canada. There is a regional AMS program governed by the pharmaceutical and therapeutic committee of the health authority.	Seven nurses and two physicians who are part of the ICU staff.	To effectively promote AMS in the ICU, perceived opportunities and barriers must be considered. To facilitate AMS activities, feasible interventions that local ICU staff would accept should be implemented in collaboration with the ICU team.
Nurses' perceptions of the potential evolution of their ole in antibiotic stewardship in nursing homes: a French qualitative study<br><br>20 Embase	Bridey et al. 2023<br>France<br><br>(Céline Bridey, Gaëlle Le Dref, Aurélie Bocquier, Stéphanie Bonnay, Céline Pulcini and Nathalie Thilly	Semi-structured face-to-face individual interviews with Nursing home nurses (NHNs) and one focus group with advanced practice registered	To explore French nurses' perceptions on ABS, current and future potential roles in ABS in nursing homes, as well as facilitators and barriers.	French nursing home.	Twenty NHNs: nineteen women, and eight are 35–44 years old. thirteen are nurses, and the others are coordinating NHNs. Half has been working in a nursing home for less than 5 years.	There is a need to strengthen nurses' participation in ABS in NHs: to invest their ABS role, to make available guidelines on ABS in NHs, to promote the role of NHNs in ABS, to provide them

	France 2023)	nurses (APRNs).			Seven APRNs participated in the focus group, including six women. Three had experience in geriatric care and the three APRN specialties were represented.	with infection management training and tool, and to strengthen multidisciplinary work.
